# Supplementary material for: Clonorchis sinensis on the prognosis of patients with spontaneous rupture of Hepatocellular Carcinoma: An inverse probability of treatment weighting analysis
Source: PLoS Negl Trop Dis. 2024 Feb 21;18(2):e0011987. doi: 10.1371/journal.pntd.0011987 (PMC10911612; doi:10.1371/journal.pntd.0011987)
Supplement: Attachment — Submitted filename: Comment for authors_PNTD-D-23-00960.pdf [file pntd.0011987.s001.pdf]

Reviewer comment

The manuscript entitled "*Clonorchis sinensis* on the prognosis of patients with spontaneous rupture of Hepatocellular Carcinoma: an inverse probability of treatment weighting analysis" is interesting work, but there are major points need to be addressed before publish as followed;

1. It would be better if the authors can provide the information of IPTW in the introduction part, including the advantages, and how it important and improve the result or previous reports.
2. This finding did not show the significant data of *C. sinensis* and no *C. sinensis* group when no using IPTW, could you please explain and discussion in this point.
3. Please *Italics* for the word "*C. sinensis*" in the text.
4. To obvious, please describe separately between the baseline characteristics and the result that comparison of before and after using IPTW. Importantly, please use Chi-square to compare the different between before and after using IPTW. Could you please explain more about the statistical analysis of this part?
5. Please elucidate how srHCC differ from HCC, what is the significant point to study?
6. Please add more risk factors of HCC and/or srHCC, relationship of HBV, HCV and *C. sinensis* with HCC.
7. It would be good if the author can add more discuss about *C. sinensis* in HCC when compare with srHCC.
