# Supplementary material for: Clonorchis sinensis on the prognosis of patients with spontaneous rupture of Hepatocellular Carcinoma: An inverse probability of treatment weighting analysis
Source: PLoS Negl Trop Dis. 2024 Feb 21;18(2):e0011987. doi: 10.1371/journal.pntd.0011987 (PMC10911612; doi:10.1371/journal.pntd.0011987)
Supplement: Attachment — Submitted filename: Response.docx [file pntd.0011987.s002.docx]

Reviewer #1

1, "Study Population" should be re-written like this: Of 328 patients with ruptured HCC, 171 were excluded (17 with previous treatment, 95 receiving TAE or TACE, 33 without treatment, and 26 without complete follow up data) is written on line 110-112.

We compared the general information of the 171 excluded patients and found that they were generally balanced between the two groups. Because these 171 cases do not require statistical analysis, they are not included in the article.

2, line250-253 have discuss the potential bias

3, “*C. sinensis* and HBV co-infection Prognostic analysis” on line 224-230 of the RESULT and on line277-285 in Discussion have been added the analysis of co-infection of HBV and *C. sinensis.*

Reviewer #2

1, We have some factors in this article, including tumore size, Edmondson,Child Pugh and so on. Chemotherapy is a very important factor, The reason we did not have statistics is that many patients did not choose to do chemotherapy.

2, “*C. sinensis* and HBV co-infection Prognostic analysis” on line 224-230 of the RESULT and on line277-285 in Discussion have been added the analysis of co-infection of HBV and *C. sinensis.*

3, We have double checked the OS before IPTW and the result is no problem. Perhaps the reason is that there are too few medical records of the *C. sinensis* group.

4, On line 148-150 have described the treatment of *C. sinensis* after postoperative.

5, *C. sinensis* have been changed to italics

Reviewer #3

1, On line 90-97 have provide the information of IPTW

2, On line 265-266 have discussed the point

3, *C. sinensis* have been changed to italics

4, The Table 1 of Result have describe the baseline characteristics

5, On line 86-89 has already explained the importance of studying ruptured hepatocellular carcinoma

6, “*C. sinensis* and HBV co-infection Prognostic analysis” on line 224-230 of the RESULT and on line277-285 in Discussion have been added the analysis of co-infection of HBV and *C. sinensis.*

7, On line 257-262 have discussed the point
