# Supplementary material for: Clonorchis sinensis on the prognosis of patients with spontaneous rupture of Hepatocellular Carcinoma: An inverse probability of treatment weighting analysis
Source: PLoS Negl Trop Dis. 2024 Feb 21;18(2):e0011987. doi: 10.1371/journal.pntd.0011987 (PMC10911612; doi:10.1371/journal.pntd.0011987)
Supplement: Attachment — Submitted filename: Response2.docx [file pntd.0011987.s003.docx]

Reviewer #2:

1, All variables in Table 1 are binary variables. We only list one variable of the binary variables, because we don't want the table to be too large. For example, gender and Age in the table should be

Male	103 (81.7)	30 ( 96.8)

Female	23 (18.3)	1( 3.2)

Age (≤50 years)	68 (54.0)	14 ( 45.2)

Age (＞50 years)	58 (46.0)	17 ( 54.8)

2, *C. sinensis* have been corrected on line 111，131，224 and 249

Reviewer #3:

1, The approval ethics number is on line 114.
